# Surgical Cryoablation of Drug Resistant Ventricular Tachycardia and Aneurysmectomy of Postinfarction Left Ventricular Aneurysm

**DOI:** 10.1155/2014/207851

**Published:** 2014-08-14

**Authors:** Marek Pojar, Jan Harrer, Nedal Omran, Martin Vobornik

**Affiliations:** Department of Cardiac Surgery, Faculty of Medicine and University Hospital, Charles University in Prague, Sokolska 581, 500 05 Hradec Kralove, Czech Republic

## Abstract

Heart failure is usually associated with left ventricle remodelling, wall thickening, and worsening of the systolic function. Ventricular tachycardia is a common and a negative prognostic factor in patients with endocardial scarring following myocardial infarction and aneurysm formation. The authors present a case of a 51-year-old man with ischemic heart disease, who suffered myocardial infarction four years ago. The patient was admitted to the hospital with sustained ventricular tachycardia despite maximal pharmacotherapy and also underwent unsuccessful percutaneous radiofrequency ablation in the right ventricle. Transthoracic echocardiography revealed left ventricle dysfunction with ejection fraction of 25%, aneurysm of the apex of the left ventricle with thrombus formation inside the aneurysm. Surgical therapy consisted of the cryoablation applied at the transitional zone of the scar and viable tissue and the resection of the aneurysm. The patient remained free of any ventricular tachycardia four months later.

## 1. Introduction

Heart failure in patients with ischemic heart disease is associated with increased mortality. The progression of heart failure is associated with left ventricle remodelling, with increased left ventricle end-diastolic and end-systolic volumes, wall thickening, and worsening of the systolic function. Left ventricular dilatation is a predictor of increased mortality in heart failure [1].

Several surgical procedures have been developed to counteract the ventricular remodelling process. Surgical ventricular restoration (SVR) involves resection of scar, septal exclusion, cavity reduction by endoventricular patch, and complete coronary grafting.

Ventricular tachycardia (VT) is a common and a negative prognostic factor in patients with postinfarction ventricular dilatation and aneurysm. This is of particular importance in patients with endocardial scarring following myocardial infarction.

The treatment of postinfarction VTs with antiarrhythmic drug therapy and implantable automatic defibrillators also includes different surgical procedures such as endocardial resection of the scar or endocardial cryoablation.

In this paper we present successful surgical treatment of sustained VT in patient with postinfarction left ventricle aneurysm complicated with thrombus formation.

## 2. Case Report

The authors present a case of a 51-year-old man with ischemic heart disease who suffered acute transmural myocardial infarction (anteroapicoseptolateral localisation) in his history, with delayed percutaneous angioplasty and stent implantation into the left anterior descending branch. Four years later, the patient (NYHA III) was admitted to the hospital with sustained ventricular tachycardia. He underwent percutaneous radiofrequency ablation in the right ventricle and implantation of the ICD (Virtuoso VR, Medtronic, Inc., Minneapolis, USA). In the next period the patient suffered repeat sustained monomorphic VT resistant to maximal pharmacotherapy with amiodarone (1200 mg/day; Sedacoron, Lek Pharmaceuticals d.d., Ljubljana, Slovinsko), prajmaline (80 mg/day, Neo-Gilurytmal, Pharmaselect International Beteiligungs GmbH, Vienna, Austria), or ajmaline (25–50 mg intravenous; Gilurytmal, Carinopharm GmbH, Elze, Germany).

**Figure 1:**
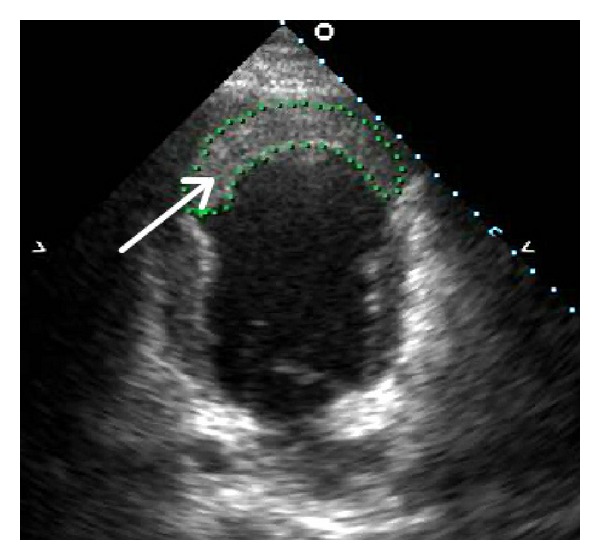
Preoperative echocardiography shows left ventricle aneurysm with the thrombus formation (arrow).

Transthoracic echocardiography revealed left ventricle dysfunction with ejection fraction of 25%, aneurysm of the apex of the left ventricle with thrombus formation inside the aneurysm (Figure 1).

**Figure 2:**
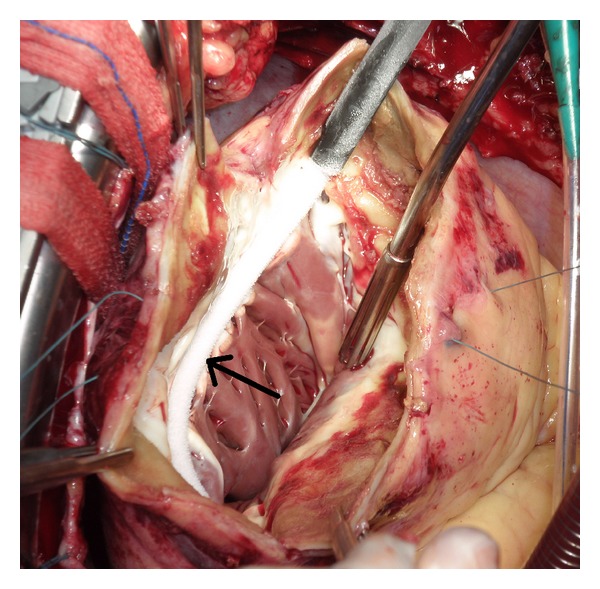
Perioperative view shows the left ventricle. After the left ventricular aneurysm was opened the transitional zone was identified. Encircling cryoablation of transitional zone between the scar and viable tissue (arrow).

The surgery procedure was carried out via median sternotomy. Cardiopulmonary bypass was established via cannulation of the ascending aorta and right atrium. The aorta was cross-clamped, and myocardial protection was achieved with intermittent cold antegrade blood cardioplegia. The left ventricle was incised parallel to the interventricular septum and the left anterior descending artery. The interventricular clots were removed. Continuous encircling linear cryolesions (CryoICE, AtriCure, Ohio, USA) were applied at the transitional zone of the scar and viable tissue (Figure 2). The left ventricle restoration was performed according to the procedure described by Dor. A purse-string suture was placed around the circumference of the scar at the transition zone and tied down to determine the size of the ventricle diameter. A prosthetic patch was then secured over ventricular opening and the free wall edges were closed over the patch. The postoperative course was uncomplicated and the patient was discharged 15 days later. Only *β*-blocker (metoprolol, Betaloc ZOK, AstraZeneca, Great Britain) was given in the postoperative period. In the period of four-month followup no recurrent ventricular tachycardia was detected. According to the ICD no VT was detected in the follow-up period, and only paroxysmal supraventricular tachycardia was detected. Transthoracic echocardiography showed improvement in the ejection fraction of the left ventricle (45%) and no signs of thrombus.

The review of the clinical information was approved by the Clinical Ethics Committee of the University Hospital in Hradec Kralove.

## 3. Discussion

The association between VT and postinfarction left ventricular aneurysm has been noted since the 1930s. Considering the fact that ventricular arrhythmia is responsible for more than 50% of death in remodelled ventricles after myocardial infarction, surgical treatment was attempted [1]. Over the past decades, surgical interventions for VT have become safer. However, the number of patients considered for surgery decreased. This can be attributed to the increased number of performed percutaneous coronary interventions, percutaneous radiofrequency ablations, and indications for implantable cardioverter-defibrillator, which may take part in decreasing the incidence of postinfarction left ventricle remodelling, aneurysm formation, and subsequently VT incidence. Nevertheless, surgical ablation combined with aneurysm resection and myocardial revascularization remains the treatment of choice for a selected population of patients.

Monomorphic ventricular tachycardia could be induced in the presence of arrhythmogenic substrate such as a left ventricular aneurysm or recent myocardial infarction. In these patients surgical revascularization alone will most likely fail to prevent the incidence of postoperative ventricular arrhythmia, especially in the presence of poor left ventricular function.

Left ventricular aneurysmectomy to treat drug-resistant VT was described in 1959 by Couch [2]. Guiraudon et al. introduced encircling endocardial ventriculotomy in 1978 [3]. Later, in 1979, Josephson et al. described the subendocardial resection procedure [4]. However, these procedures were associated with high mortality and limited success rate. That may be because most VTs are provoked from the border of the aneurysm, which is rarely excised during operation. Extensive ablation procedure using cryoablation energy has been recently introduced into the clinical practice and proved its efficacy in suppressing the incidence of postoperative VT.

Among applied several surgical procedures, two major approaches have become prevalent. These are ablation or resection of arrhythmogenic foci performed after precise either intraoperatively or percutaneously mapping or more extensive surgical ablation procedures without mapping (including cryoablation or thermoexclusion by laser).

Generally, map-guided procedures have been used for surgical treatment of VT. Ostermeyer et al. demonstrated good long-term results of electrophysiologically guided resection for VT with survival rate of 75% and 45% at 1 year and 5 years, respectively [5]. However, there is an ongoing debate on the use of intraoperative cardiac mapping.

The results yielded from other studies reported favourable outcomes of nonguided methods [6]. In the study of Frapier et al. the freedom from VT was 77% at 5 and 7 years after surgery [7]. Demaria et al. reported long-term results of encircling cryoablation in a cohort of 52 patients without mapping. During their 14 years of followup they reported 86% rate of freedom from VT or sudden cardiac death [8].

Sartipy et al. from The Karolinska group in Stockholm presented 53 patients who underwent the Dor procedure with nonelectrophysiologically guided subtotal endocardectomy and cryoablation for VT at the transition zone of the scar and viable tissue. Early mortality was 3.8%. Overall survival was 94%, 80%, and 59% at 1 year, 3 years, and 5 years, respectively. Freedom from spontaneous VT after a mean follow-up period of 3.7 ± 2.0 years was 90%. Postoperative VT suppression was similar in patients with preoperative spontaneous or inducible VT [9].

Wellens et al. have published their experience with endoaneurysmorrhaphy and cryoablation without intraoperative mapping in a population of 31 patients with hospital mortality rate of 6.5%. Postoperative electrophysiologic study revealed freedom from VT induction in 25 patients and inducible VT in five patients [10].

These studies show that there is a subset of patients who remarkably benefits from the combined approach for treatment of postinfarction VT including aneurysmectomy of postinfarction left ventricular aneurysm and extended blind cryoablation without perioperative mapping. It is a simple surgical technique, reproducible in most of the cardiac centres where a cryosource is available. It offers excellent arrhythmia control and satisfactory clinical outcomes.

## 4. Conclusion

Cryoablation without mapping and anerurysmectomy offers good arrhythmia control and long-term clinical outcome in patients presenting with VT arising from post-myocardial infarction aneurysm.
